# Influence of Boron on the Microstructural Evolution, Impact and Creep Properties Stability of IN718 Superalloy During Long-Term Aging

**DOI:** 10.3390/ma19061152

**Published:** 2026-03-16

**Authors:** Zhiyuan Wang, Yingjie Liu, Ning An, Jia Man, Xin Xin, Jianyong Li, Maocheng Ji, Wenru Sun

**Affiliations:** 1 Key Laboratory of High Efficiency and Clean Mechanical Manufacture, Ministry of Education, School of Mechanical Engineering, Shandong University, Jinan 250061, China; 2 Institute of Metal Research, Chinese Academy of Sciences, Shenyang 110016, China; yjliu20s@imr.ac.cn (Y.L.);; 3 Beijing Beiye Advanced Materials Co., Ltd., Beijing 100192, China

**Keywords:** IN718 superalloy, boron, long-term aging, microstructure stability, mechanical properties

## Abstract

The impact of boron (B) on the microstructure evolution and stabilization of mechanical properties in the IN718 superalloy during aging at 680 °C for 3000 h is investigated. The results indicated that B had negligible effects on grain size and the intragranular γ″ phase growth. In contrast, it effectively suppressed the precipitation and growth of the δ phase during long-term aging, which is attributed to grain boundary segregation of B that retards the diffusion of alloying elements. Adding B could improve the impact toughness and stability of the creep properties of the alloy. The primary mechanism is that the addition of B enhances grain boundary cohesion and suppresses the coarsening of the δ phase, while the beneficial effect of B on mechanical stability becomes negligible during the later stages of aging, as the severe coarsening of grain boundary phases offsets the enhanced grain boundary cohesion resulting from B segregation. Furthermore, the presence of slip bands was observed in the creep deformation mechanism of B-added alloys, which is likely attributable to B promoting dislocation slip at grain boundaries. With prolonged aging time, the dominant creep deformation mechanism in the B-modified alloy shifts from being primarily governed by twinning and dislocation slip to a mechanism involving twinning, stacking fault shearing γ″ phase, and dislocation slip.

## 1. Introduction

The IN718 superalloy is a critical material for manufacturing high-performance components in aero-engine turbines, owing to its exceptional high-temperature strength, corrosion resistance, and mechanical stability under demanding operating conditions [1,2,3,4]. Its outstanding properties stem from a complex microstructure that comprises the γ-matrix, ordered γ″ and γ′ phases, along with the δ phase [5]. With rising turbine entry temperatures in advanced aero-engines, hot-section components like blades and turbine discs experience significantly higher operating temperatures. This trend severely limits the potential use of the IN718 superalloy in next-generation engines, since the alloy undergoes rapid microstructural degradation above 650 °C [6,7]. Principal forms of degradation include the transformation of the strengthening γ″ phase into the δ phase, rapid coarsening and coalescence of the δ phase, and precipitation of topologically close-packed (TCP) phases. These microstructural changes collectively lead to a significant deterioration in the mechanical stability of the IN718 superalloy [8,9,10]. Adding trace elements offers a promising and economical way to achieve a balance between the high-temperature strength and other performance characteristics of alloys. The trace element B is generally recognized to improve the mechanical properties of superalloys, especially their stress rupture and creep properties [11,12,13]. However, the mechanism underlying this improvement remains unclear, and various potential explanations have yet to be fully explored.

B is one of the most widely utilized beneficial microalloying elements in superalloys and is incorporated in the vast majority of wrought and cast superalloys [14,15,16,17]. Even single-crystal superalloys often contain a small quantity of B, which serves to strengthen potential low-angle boundaries [18]. In 1958, Decker et al. [19] serendipitously discovered that B from the crucible material could improve the stress rupture properties of alloys. Subsequent research confirmed that a B content range of 0.002 to 0.025 wt.% produced the most significant enhancement in creep and stress rupture properties [20,21,22]. Three-dimensional atom probe tomography has been used to systematically reveal the microscopic distribution of B in superalloys. This has shown that B segregates at grain boundaries and phase interfaces (such as M_23_C_6_/γ and γ′/M_5_B_3_), while being depleted in the γ matrix [23,24]. Furthermore, Antonov et al. [25] revealed B enrichment along dislocation lines within the matrix and at grain boundaries in a laser-remelted Ni-based superalloy. Currently, the beneficial effects of B in superalloys are primarily mediated by its segregation to grain boundaries and associated defects, resulting in the following principal effects: Firstly, B enhances grain boundary cohesion by strengthening metallic bonds and reducing boundary energy [26,27,28]. Secondly, it competitively suppresses the segregation of harmful impurities (O, S, and H), improving resistance to intergranular cracking [29,30]. Thirdly, the segregation of B promotes the formation of serrated grain boundaries, which inhibit grain boundary sliding and impede cavity nucleation and growth during fatigue, thereby enhancing alloy plasticity [31,32]. Finally, B segregation at interphase boundaries alters the morphology, quantity, and distribution of grain boundary phases, which further improves stress rupture and creep properties [20,21,33,34].

The microstructural stability of the IN718 superalloy is crucial for retaining its mechanical properties during long-term, high-temperature service [35,36]. To date, studies on B in the IN718 superalloy have primarily focused on its role as a grain boundary strengthener under standard heat treatment. However, its effects on microstructural evolution and the stability of mechanical properties remain largely unexplored. Therefore, this study systematically investigates the influence of B on the microstructural evolution, stability of mechanical properties, and deformation mechanisms of the IN718 superalloy during long-term aging. A combined analysis of impact toughness and creep properties was conducted to better clarify the relationship between microstructural changes and mechanical performance. The results offer crucial insights into the strengthening mechanisms of B in superalloys and provide essential guidelines for optimizing the IN718 superalloy for advanced high-temperature applications.

## 2. Experimental Procedure

### 2.1. Material

The IN718 superalloy used in this study was prepared by vacuum induction remelting (VIR) with a nominal composition (wt.%) of 19.12% Cr, 52.3% Ni, 5.25% Nb, 3.08% Mo, 1.03% Ti, 0.72% Al, and the balance Fe. To minimize compositional variations, the IN718 superalloy was remelted. During this process, B was added in varying amounts for subsequent microstructural and mechanical properties analysis. B was introduced via a Ni-B master alloy with a B content of 18.3%. The B content in the IN718 superalloys was quantified by inductively coupled plasma atomic emission spectrometry (ICP-AES). Based on the measured concentrations (0, 0.0026, and 0.0065 wt.%), the alloys are referred to as B0, B1, and B2, respectively.

### 2.2. Heat Treatment

A multi-step homogenization treatment (1120 °C/30 h + 1160 °C/30 h + 1190 °C/40 h) was applied to the ingots to dissolve the δ phase, Laves phase, and elemental segregation in the cast structure. Following homogenization, the ingots were forged at 1120 °C into 40 mm square billets. Subsequently, the billets were rolled into round bars with a diameter of 13 mm through multiple passes. All hot-rolled bars underwent direct aging (DA) heat treatment: 720 °C × 8 h, cooled to 620 °C at 50 °C/h, held at 620 °C × 8 h, followed by air cooling. Finally, all hot-rolled bars underwent long-term aging at 680 °C for 500 h, 1500 h, and 3000 h for analyses of microstructure and mechanical properties. Long-term aging was conducted in air using a muffle furnace with a heating rate of 10 °C/min. The temperature was maintained at 680 ± 2 °C throughout the exposure period, as monitored by a thermocouple attached to the sample. After aging, samples were air-cooled to room temperature.

### 2.3. Experimental Techniques

The microstructure of DA-state and aged-state alloys were characterized by an INSPECT F50 field emission scanning electron microscope (SEM) equipped with an electron backscatter diffraction (EBSD) detector. The samples for SEM were ground, polished with 2.5 mm diamond abrasive, and chemically etched using a solution with 5 g CuCl_2_ + 100 mL HCl + 100 mL C_2_H_6_. The EBSD samples were electrophished in a 10% perchloric acid alcohol solution at 20 V for 30 s. The EBSD test was conducted at 20 kV with a scan step size of 0.1 μm, and the EBSD data were processed and analyzed using AZtecCrystal software (Version 3.1). The evolution of precipitate phases in the alloys during aging was characterized using an FEI Talos F200X transmission electron microscope (TEM) (Thermo Fisher Scientific, Waltham, MA, USA). The creep deformation microstructure at different aging times was further examined by high-angle annular dark-field (HAADF) imaging and high-resolution TEM (HRTEM). The TEM samples were mechanically ground to below 50 µm in thickness and punched into 3 mm discs, and then the thin foils were prepared in a twin-jet electropolishing with a solution of 10% perchloric acid in alcohol solution at 20 V and −23 °C.

Samples were taken from alloy bars with different B contents and machined into standard impact specimens with KU_2_-type notches. The dimensions of the impact specimen were 10 mm in width and 55 mm in length. Room-temperature impact tests were conducted on a SANS-ZBC2452-C impact testing machine (MTS, Shenzhen, China) in accordance with the GB/T 229-2020 standard [37]. At least two specimens were tested for impact performance under each condition. Creep test specimens were machined to standard dimensions with a gauge length of 27 mm and a diameter of 5 mm. Creep tests were carried out at a CSS-3910 computer-controlled electronic creep testing machine (Changchun Research Institute for Mechanical Science Co., Ltd. (CCTM); Changchun, Jilin Province, China) under the conditions of 650 °C/725 MPa. To ensure statistical reliability, at least two creep specimens were tested for each condition.

## 3. Results and Discussion

### 3.1. Effect of B on the Microstructure

#### 3.1.1. DA-State Alloys

**Figure 1:**
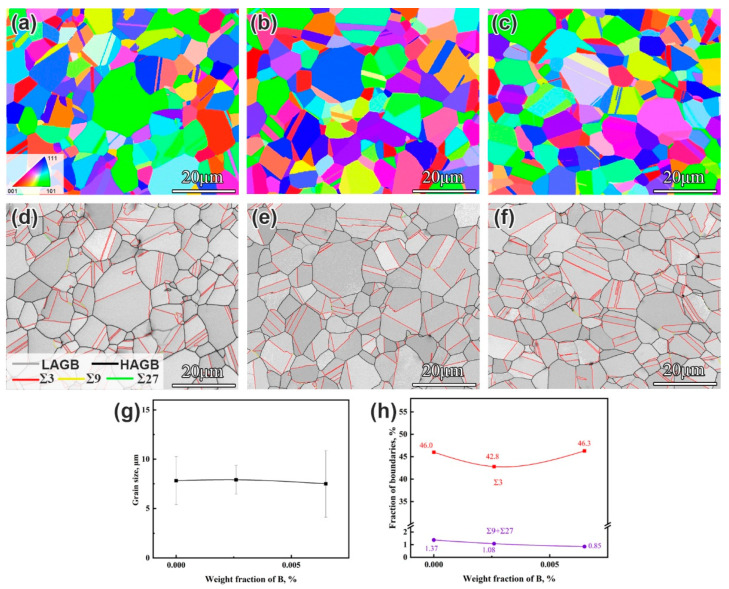
(**a**–**c**) IPF maps of the B0, B1, and B2 alloys, respectively; (**d**–**f**) the corresponding grain boundary network maps of the B0, B1, and B2 alloys, respectively; and (**g**,**h**) the change in grain size and characteristic boundaries fraction with B contents in the alloys, respectively.

Figure 1 shows the EBSD analysis of grain structure characteristics in the alloys with different B contents after DA treatment. The inverse pole figure (IPF) maps reveal that all the alloys are composed of equiaxed grains with no preferred orientation. The distribution maps of characteristic grain boundaries reveal that all alloys contain a high fraction of high-angle grain boundaries (HAGBs) and low ΣCSL (Σ ≤ 27) boundaries. To describe the effect of B on grain structure characteristics more specifically, the size of the grains and the fractions of the characteristic grain boundaries were analyzed statistically (Figure 1g,h). The grain size of the DA-state alloys shows no significant dependence on the B contents, maintaining an average of about 7 µm. Moreover, the fractions of characteristic grain boundaries show negligible variations across different B contents, indicating that B has a limited influence on the characteristic grain boundaries in the IN718 superalloy.

**Figure 2:**
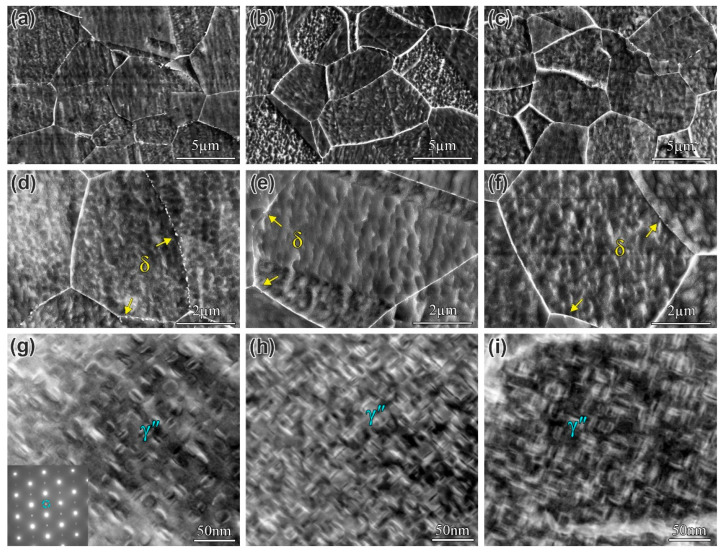
(**a**–**c**) The microstructure morphology of the B0, B1, and B2 alloys, respectively; (**d**–**f**) the morphology of δ phase in the B0, B1, and B2 alloys, respectively; and (**g**–**i**) TEM images showing the morphology of the γ″ phase of B0, B1, and B2 alloys, respectively.

The microstructure morphology of the alloys after DA treatment is shown in Figure 2. The alloys with different B contents displayed relatively smooth grain boundaries, with fine δ phase particles precipitated only at certain grain boundaries. Higher-magnification SEM images (Figure 2d–f) show that the amount and size of the δ phase are reduced at the grain boundaries of the B-modified alloys compared to the B0 alloy. The intragranular γ″ phases in the three DA-state alloys were characterized by TEM (Figure 2g–i), and it can be observed that the γ″ phase exists in three distinct variants. Statistical analysis of the major axis length of the γ″ phase shows that the average values for the B0, B1, and B2 alloys are 12.65 nm, 11.75 nm, and 11.36 nm, respectively. These results suggest that the influence of B on the size of the γ″ phase is limited.

#### 3.1.2. Aged-State Alloys

**Figure 3:**
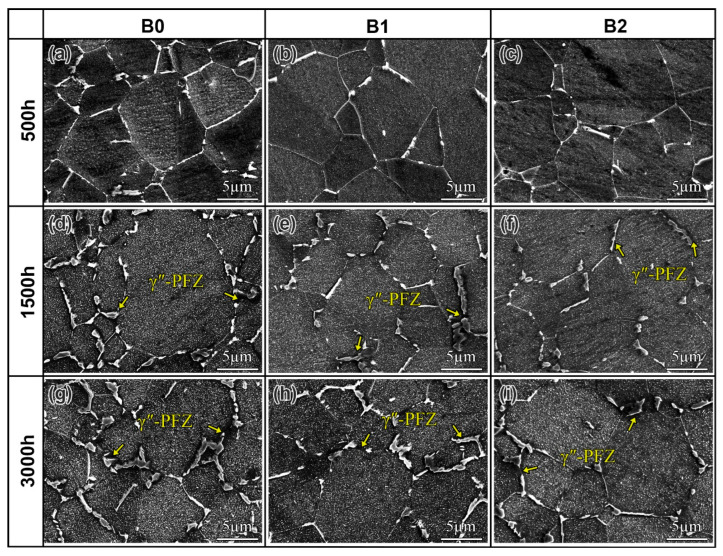
The evolution of the δ phase in the alloys with varying B contents during long-term aging at 680 °C: (**a**–**c**) B0, B1, and B2 alloys aging for 500 h, respectively; (**d**–**f**) B0, B1, and B2 alloys aging for 1500 h, respectively; and (**g**–**i**) B0, B1, and B2 alloys aging for 3000 h, respectively.

The evolution of the δ phase was investigated in alloys with varying B contents during long-term aging at 680 °C for 3000 h, as shown in Figure 3. After 500 h, the δ phase in the B0 alloy coarsened markedly, forming a continuous film along the grain boundaries. In contrast, the B1 and B2 alloys retained a dispersion of δ particles. After aging for 1500 h, the grain boundary δ phase coarsened significantly in alloys with different B contents. Notably, the B-containing alloys exhibited a lower δ phase quantity compared to the B0 alloy. Furthermore, the γ″ phase free zones (PFZs) began to form adjacent to the grain boundary δ phase in all alloys, as indicated by the yellow arrows in Figure 3d–f. After 3000 h of aging, the grain boundaries in the B0 alloy were almost entirely covered by the δ phase, whereas the B1 and B2 alloys contained a lower amount of δ phase. Concurrently, the areas devoid of the γ″-PFZs expanded in all alloys. In summary, the addition of B can inhibit the coarsening of the δ phase during long-term aging. In general, the coarsening of the δ phase is mainly controlled by the diffusion of Nb [38,39]. The segregation of B at grain boundaries occupies vacancies and lattice defects [40]. This process slows the diffusion of δ phase-forming elements, thereby suppressing the coarsening and agglomeration of the δ phase along the boundaries [20,41]. Consequently, the absence of B at the grain boundaries in the B0 alloy results in continual precipitation and coarsening of the δ phase during long-term aging.

**Figure 4:**
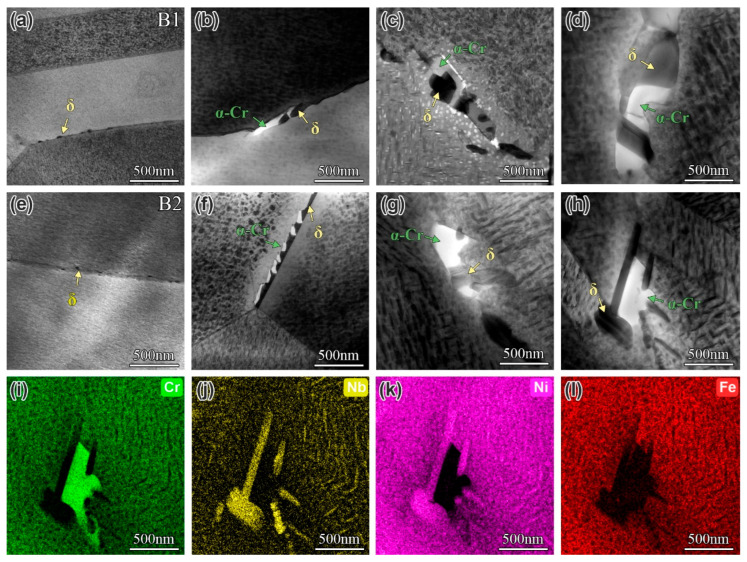
The evolution of α-Cr phase at grain boundary in the alloys with different B contents during long term aging: (**a**–**d**) B1 alloy aging for 0 h, 500 h, 1500 h, and 3000 h, respectively; (**e**–**h**) B2 alloy aging for 0 h, 500 h, 1500 h, and 3000 h, respectively; and (**i**–**l**) the corresponding EDS mapping results of α-Cr and δ phases in (**h**).

Previous studies have also reported the formation of α-Cr phase in the IN718 superalloy during extended aging [42,43]. The effect of B contents on the precipitation behavior of the α-Cr phase was investigated by TEM characterization of B1 and B2 alloys aged for various times, as shown in Figure 4. In the DA (0 h) state, the microstructure contained finely dispersed δ-phase particles (40–50 nm) along grain boundaries, while no α-Cr phase was detected (Figure 4a,e). After 500 h of aging, an irregular blocky α-Cr phase precipitated, alternating with the δ phase, with sizes ranging from 120 to 380 nm in the alloys. Upon further aging, a commensurate increase was observed in both the quantity and dimensions of the α-Cr phase. Following 3000 h of aging, the α-Cr phase attained a size of approximately 500 nm in the B-containing alloys. A representative TEM-EDS analysis of the B2 alloy aged for 3000 h is shown in Figure 4i–l. The results indicate that the α-Cr phase is primarily enriched in Cr and depleted in Nb and Ni. Conversely, the δ phase is enriched in Nb and Ni and depleted in Cr. Additionally, both phases were found to be depleted in Fe. This complementary distribution suggests elemental partitioning between the two phases, which further demonstrates that the growth of the δ phase occurs through the incorporation of Nb and the rejection of Cr, thereby facilitating the nucleation of the α-Cr phase during the aging process. Compared to the previously studied IN718 superalloy (B0 alloy) [44], the B contents showed no significant effect on the precipitation and coarsening behavior of the α-Cr phase at grain boundaries. B addition is known to significantly alter the precipitation of grain boundary phases (MC, M_23_C_6_, and borides). By enriching at these phase interfaces, B interacts with TCP-forming elements, which in turn inhibits the formation and growth of deleterious TCP phases over extended aging. However, no significant segregation of B at the δ-phase interfaces in the IN718 alloy has been demonstrated; consequently, its influence on the precipitation and coarsening of the α-Cr phase is minimal. In addition, the α-Cr phase, as a TCP phase, precipitates only during aging and under stress. Its amount and size are relatively small compared to the δ phase, resulting in a minor influence of B on its precipitation behavior.

**Figure 5:**
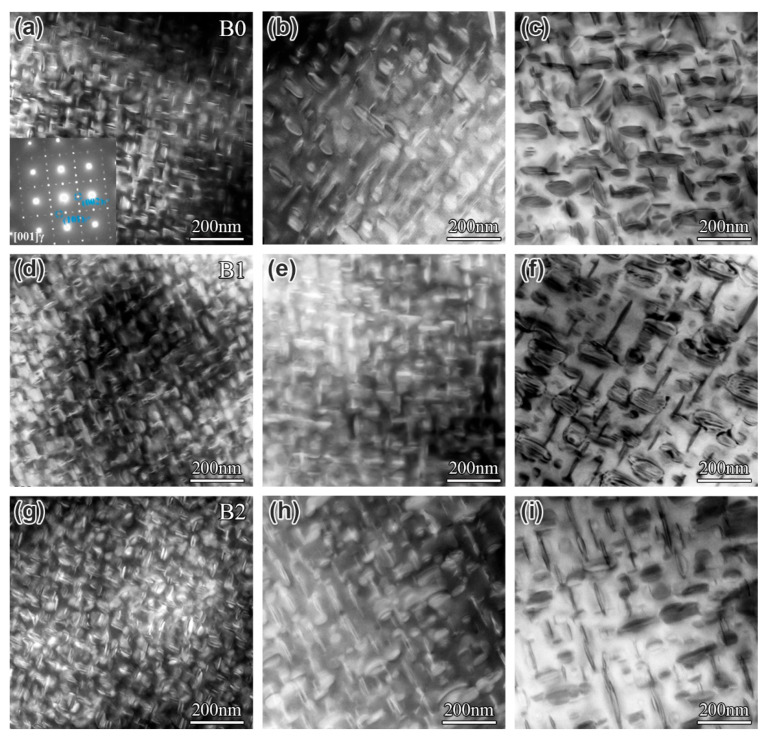
Morphology of the γ″ phase in IN718 superalloys with different B contents after aging at 680 °C for various times: (**a**–**c**) B0 alloy aged for 500 h, 1500 h, and 3000 h, respectively; (**d**–**f**) B1 alloy aged for 0 h, 500 h, 1500 h, and 3000 h, respectively; and (**g**–**i**) B2 alloy aged for 500 h, 1500 h, and 3000 h, respectively.

**Figure 6:**
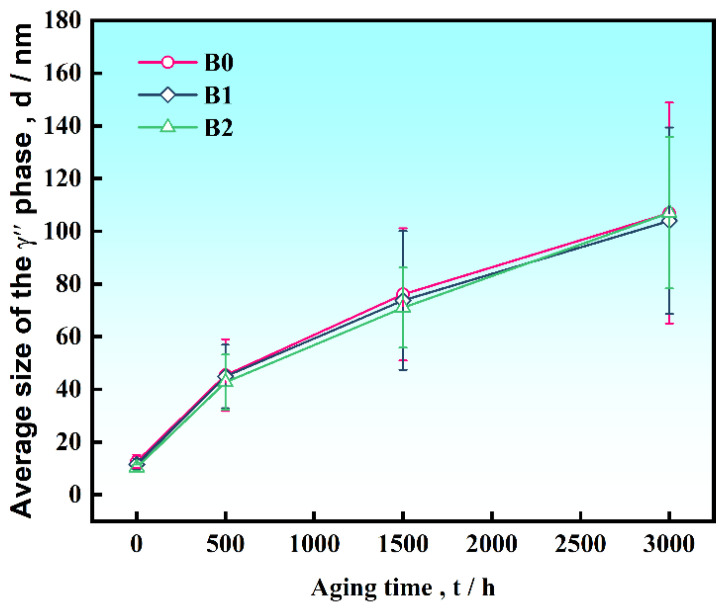
The change in γ″ phase average size with aging time in the alloys with different B contents.

In addition, the evolution of the γ″ phase in the alloys with different B contents during long-term aging was observed, as shown in Figure 5. The γ″ phase was dispersed in the matrix in a disc-shaped or lenticular morphology in all alloys. With prolonged aging time, the size of the γ″ phase gradually increased, while its number density decreased. The average diameter of the γ″ phase along the major axis in the two alloys was measured using Nano Measurer software, and the results are presented in Figure 6, which indicates that at the same aging time, the size of the γ″ phase differs only slightly among alloys with different B contents. Calculation results and TEM images revealed that the size, quantity, and morphology of the γ″ phase remained largely consistent. It is demonstrated that B addition has an insignificant effect on the coarsening of the γ″ phase in IN718 superalloys during long-term aging.

### 3.2. Effect of B on the Mechanical Properties

#### 3.2.1. Impact Properties

**Figure 7:**
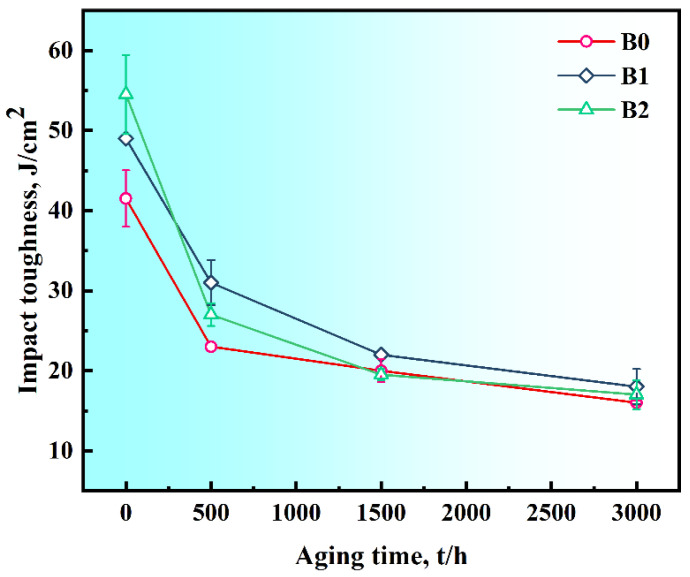
Room-temperature impact toughness of the DA alloys with different B contents during long-term aging at 680 °C.

Figure 7 presents the room-temperature impact toughness of the aged alloys with various B contents. The impact toughness of the alloys decreased sharply in the initial aging stage, and then the rate of decline gradually slowed with further aging. At 0 h aging time (DA state), the alloys showed an increase in impact toughness with B contents. The B2 alloy exhibited the highest value (54.5 J/cm^2^), which was approximately 1.3 times that of the B0 alloy (41.5 J/cm^2^). After 500 h of aging, the B-containing alloys still exhibited superior impact toughness compared to the 0B alloy. When the aging time is extended to 1500 h, the impact toughness of alloys with different B contents shows little difference. Ultimately, at 3000 h, the impact toughness values for all alloys decreased to a similar level of around 16 J/cm^2^.

**Figure 8:**
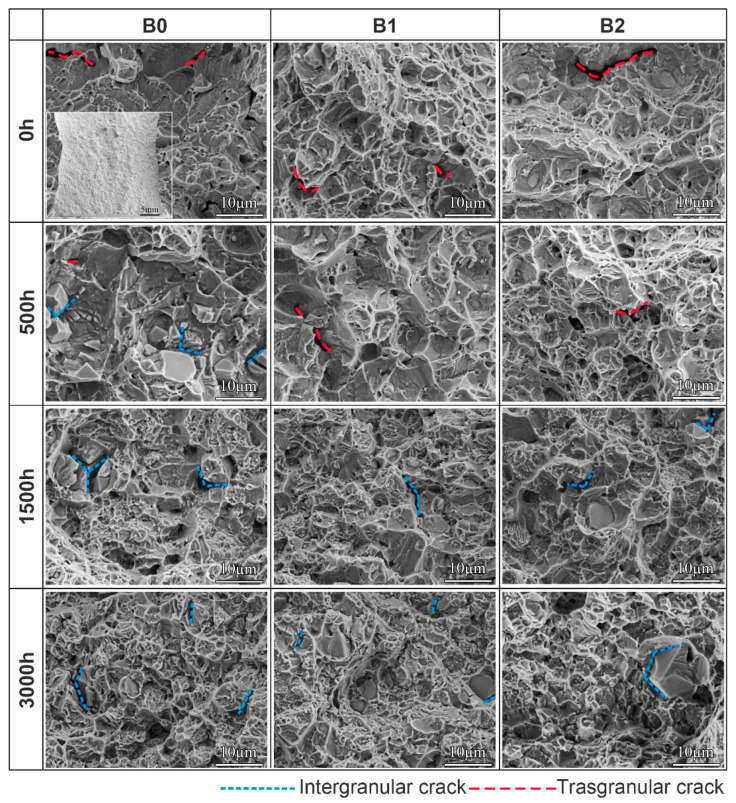
Room-temperature impact fracture morphologies of the alloys with different B contents after aging for different times.

Figure 8 shows the effect of B content on the room-temperature impact fracture morphology of long-term aged alloys. All samples exhibited a rough macroscopic fracture surface with a distinct river-like pattern, as can be seen in the inset of Figure 8. At 0 h of aging, the fracture surfaces were characterized by numerous shallow ductile dimples and a sparse distribution of transgranular cracks. No discernible variation in this morphology was observed across alloys with different B contents. Following 500 h of aging, intergranular cracks initiated in the B0 alloy, in contrast to the B1 and B2 alloys, whose fracture surfaces remained essentially the same as in the unaged condition (0 h). Further aging for up to 1500 h resulted in a progressive diminution of ductile fracture characteristics, accompanied by the emergence of intergranular cracking and quasi-cleavage facets in alloys with different B contents. After 3000 h of aging, intergranular fracture became increasingly dominant, and the impact fracture mode of all the alloys shifted to a mixed transgranular–intergranular type. In summary, during the early stages of aging, the addition of B enhances grain boundary cohesion and delays the formation of intergranular cracks. However, this beneficial effect gradually diminishes with prolonged aging time.

**Figure 9:**
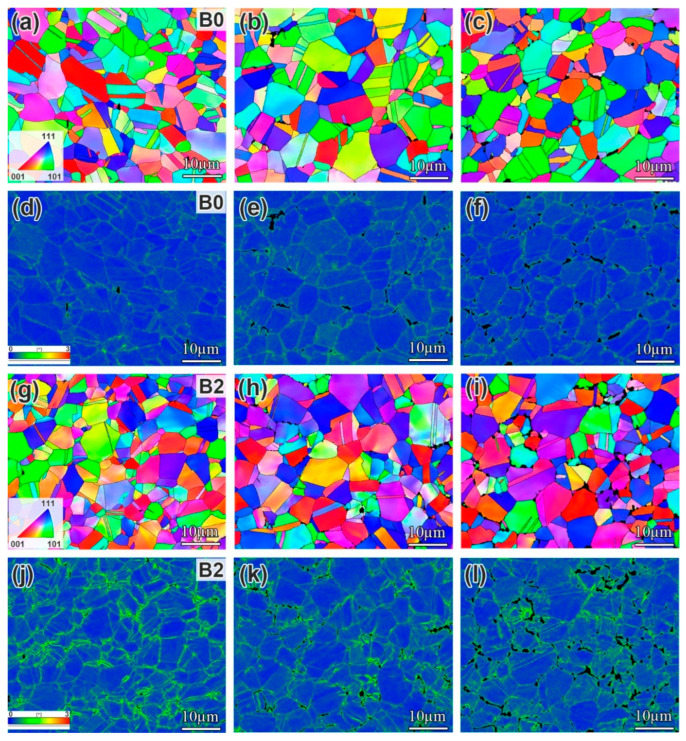
EBSD analysis of room-temperature impact fracture of alloys with different B contents after aging for different times: (**a**–**f**) the IPF maps and corresponding KAM distribution of B0 alloy after aging for 0 h, 500 h, and 1500 h, respectively; (**g**–**l**) the IPF maps and corresponding KAM distribution of B2 alloy after aging for 0 h, 500 h, and 1500 h, respectively.

EBSD analysis was conducted on the longitudinal sections of impact fractures from the B0 and B2 alloys after different aging times, as shown in Figure 9. The observed region was located near the U-notch of the impact specimen, approximately 30 μm away from the fracture surface. The IPF maps indicate that the grains in the B0 alloy exhibited no significant preferred orientation at any aging time. In contrast, those in the B2 alloy underwent progressive rotation with extended aging, showing increasingly aligned orientations along the <111> and <001> directions. It is noteworthy that at the same aging time, the kernel average misorientation (KAM) value of the B2 alloy was significantly higher than that of the B0 alloy. A higher KAM value indicates that the grains underwent a large amount of plastic deformation during impact fracture in the B2 alloy, which contributes to higher impact toughness.

According to the aforementioned results, the increase in B contents enhanced the impact toughness of the alloys before 500 h of aging. Fractographic examination reveals that after 500 h of aging, the B0 alloy exhibited intergranular fracture features, while the B-modified alloy still maintained a dimple fracture morphology. This demonstrates that B addition enhances grain boundary cohesion, thereby delaying intergranular crack initiation [45]. The strengthening is attributed to two mechanisms: B atoms fill grain boundary defects and interact with dislocations, both of which enhance cohesion [24,32]. Furthermore, B inhibits the precipitation and coarsening of the δ phase at grain boundaries. These dual effects increase intergranular cohesive strength and alleviate stress concentration, ultimately improving impact toughness. With further extended aging, the beneficial effect of B diminishes due to the rapid precipitation and coarsening of grain boundary δ phases, resulting in comparable impact toughness among alloys with different B contents [46].

#### 3.2.2. Creep Properties

**Figure 10:**
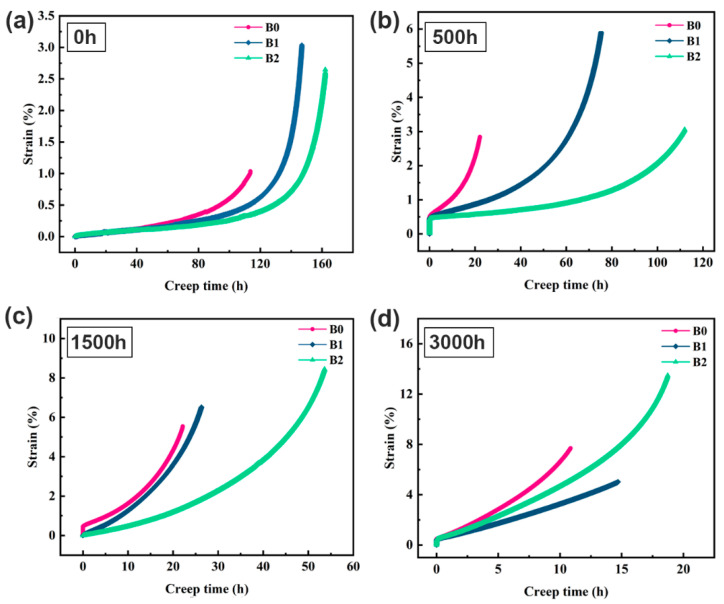
Creep curves of the alloys with different B contents at 650 °C/725 MPa after aging for various times: (**a**) 0 h, (**b**) 500 h, (**c**) 1500 h, and (**d**) 3000 h.

Figure 10 shows the creep curves of various B-content alloys under 650 °C/725 MPa after different aging times. As aging time increases, the creep life of the alloys declines monotonically. However, creep life increases with higher B content at the same aging time. After aging for 0 h, the creep lives of the B1 were 1.2 times those of the B0 alloy, and the B2 alloy demonstrates the highest creep life. After 500 h of aging, the creep life of the B0 alloy dropped significantly to approximately 25 h. Under the same condition, the B2 alloy exhibited a creep life 4.7 times that of the B0 alloy. After aging for 1500 h, the creep life of B-containing alloys decreased significantly. The lives of B1 and B2 alloys were reduced to 25 h and 52 h, respectively, but remained higher than that of the B0 alloy. After 3000 h of aging, the beneficial effect of B addition diminished, with the creep life of all alloys declining to less than 20 h. In summary, B addition contributed to improved long-term creep stability in the IN718 superalloy.

**Figure 11:**
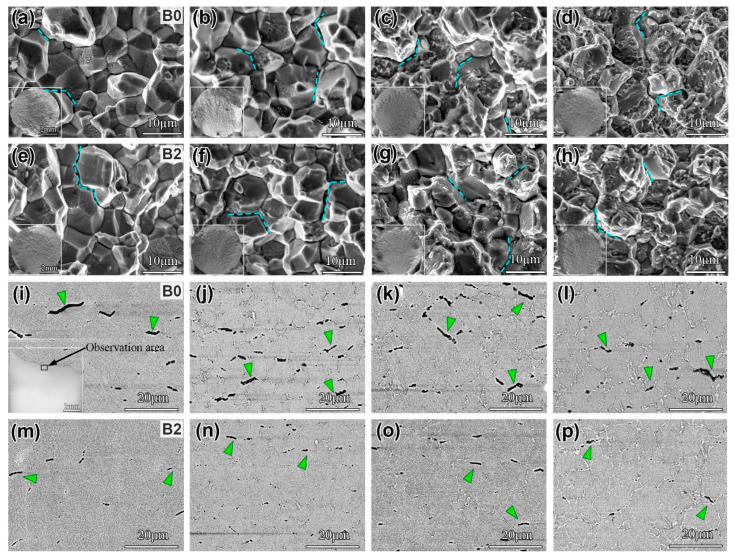
(**a**–**d**) The creep fracture surface morphology of the B0 alloy after aging for 0 h, 500 h, 1500 h, and 3000 h, respectively; (**e**–**h**) the creep fracture surface morphology of the B2 alloy after aging for 0 h, 500 h, 1500 h, and 3000 h, respectively; (**i**–**l**) the creep fracture longitudinal section of the B0 alloy after aging for 0 h, 500 h, 1500 h, and 3000 h, respectively; and (**m**–**p**) the creep fracture longitudinal section of the B2 alloy after aging for 0 h, 500 h, 1500 h, and 3000 h, respectively.

Based on the linear relationship between B content and improved creep performance, the creep fractography and longitudinal sections of B0 and B2 alloys under different aging times were investigated in this study, as illustrated in Figure 11. Macroscopic observation indicates that creep cracks originated on the surface of the specimen and spread inwards in all alloys, as illustrated in the insets of Figure 11a–h. At various aging times, the fracture surfaces exhibited numerous intergranular cracks (indicated by blue dashed lines in Figure 11), consistent with an intergranular fracture mode. Notably, the creep fracture morphology transitions from relatively smooth to distinctly rough with prolonged aging. This transition is driven by the coarsening of the δ-phase, which introduces significant stress concentrations. These elevated stress fields preferentially accelerate the nucleation, growth, and subsequent coalescence of creep cavities along the δ-phase/matrix interfaces [46,47]. The linkage of these cavities consequently initiates microcracks, which propagate to produce the characteristic progressively roughened intergranular fracture morphology. The microstructures adjacent to the fracture surface (at a distance of 30 μm from the fracture) on the creep longitudinal sections of both B0 and B2 alloys were examined, as shown in Figure 11i–p. At aging for 0 h, the B0 alloy exhibited a higher number of secondary cracks, while the B2 alloy showed fewer and discontinuous secondary cracks. After aging for 500 h and 1500 h, compared to the B2 alloy, the B0 alloy demonstrated a significantly greater number and larger size of secondary cracks. With the aging time increased to 3000 h, a corresponding decrease in the number of secondary cracks was observed in both alloys, with the B2 alloy showing a lower count. Furthermore, the stability of the alloy’s surface and its resistance to oxidation are critical for long-term performance. As demonstrated in studies on similar nickel-based systems, the interaction between plastic deformation, oxidation, and surface degradation can significantly influence crack initiation [48]. Therefore, in future work, we will further explore the effect of trace boron addition on surface stability and oxidation resistance during long-term aging.

As shown in Figure 11, at the same aging time, the number and size of cracks in the B2 alloy are both smaller than those in the B0 alloy. This difference is primarily attributed to the following factors. In the B0 alloy, the grain boundary strength decreases during high-temperature creep, making it prone to crack formation under stress [49]. Additionally, the continuously distributed δ phase along the grain boundaries induces stress concentration, which further promotes crack initiation and propagation, ultimately leading to premature fracture of the alloy. In contrast, B segregation at the grain boundaries in the B2 alloy enhances grain boundary strength and suppresses grain boundary migration during creep, thereby reducing the tendency for intergranular crack formation. Meanwhile, the B2 alloy exhibits a lower volume fraction of the δ phase, which is distributed mainly as discrete particles along the grain boundaries. The interfaces between these δ particles and the γ matrix help to alleviate stress concentration and hinder the linkage and growth of grain-boundary micropores, thus inhibiting crack propagation.

**Figure 12:**
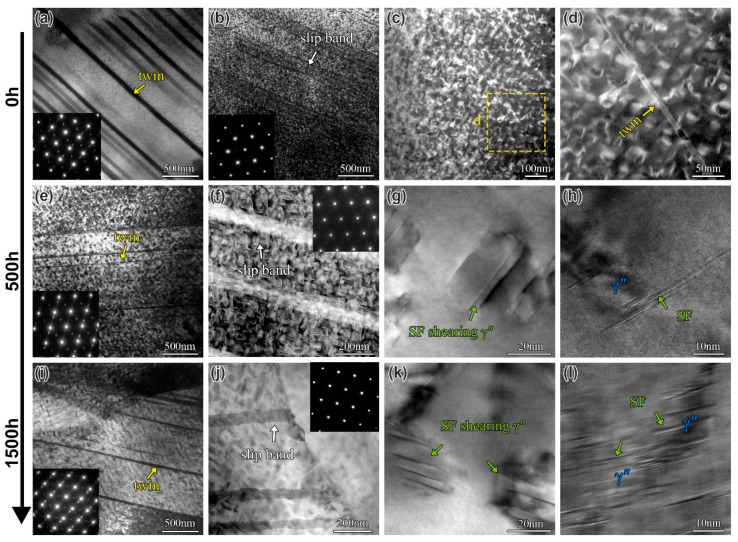
The creep deformation microstructure of B2 alloy for different aging times: (**a**,**e**,**i**) TEM images showing the twin of the alloy at 0 h, 500 h, and 1500 h, respectively; (**b**,**f**,**j**) TEM images showing the slip band of the alloy at 0 h, 500 h, and 1500 h, respectively; (**c**,**d**) TEM dark-field images showing twin shear γ matrix at 0 h; (**g**,**h**) TEM and HRETM images showing the SF shear γ″ phase at 500 h; and (**k**,**l**) TEM and HRETM images showing the SF shear γ″ phase at 1500 h.

Since the creep deformation mechanisms of the IN718 superalloy (B0 alloy) have been systematically investigated in the author’s previous studies [43,44], this work focuses primarily on examining the creep deformation microstructure of the B2 alloy under various aging conditions, as shown in Figure 12. In the B2 alloy aged for 0 h, a high density of twins and slip bands was observed (see the absence of twin spots in the SAED pattern in Figure 12b). In contrast, previous results indicated that only twins were present in the B0 alloy under the same conditions [44]. TEM dark-field images reveal that twins shear through the γ matrix but do not shear the γ″ precipitates (Figure 12c,d). This is likely due to the small size of the γ″ phase. After aging for 500 h, the primary deformation mechanisms in the B2 alloy are still dominated by twinning and slip bands. Additionally, TEM and HRTEM images show that the γ″ phase in the B2 alloy has grown slightly (by approximately 30 nm) at this stage and has begun to undergo stacking fault (SF) shear (Figure 12g,h). Following 1500 h of aging, the predominant deformation mechanisms remained twin, slip bands, and SF shearing of the γ″ phase. It is noteworthy that the γ″ phase underwent severe coarsening and was repeatedly sheared by SFs (Figure 12l).

**Figure 13:**
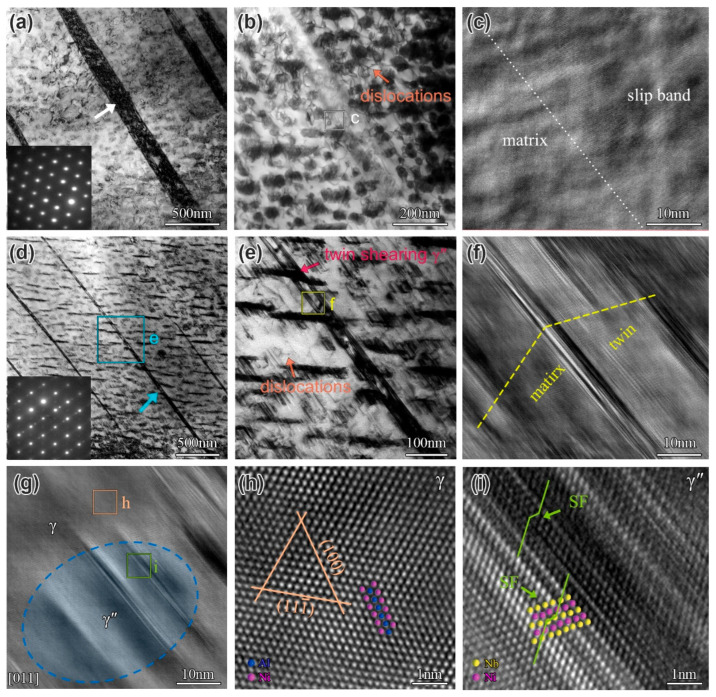
The creep deformation microstructure of B2 alloy at 3000 h: (**a**,**b**) TEM and HADDF images showing the slip band, respectively; (**c**) the corresponding HRTEM image of the area “c” in the (**b**); (**d**,**e**) TEM and HADDF images showing the twin, respectively; (**f**) the corresponding HRTEM image of the area “f” in the (**e**); (**g**) HRTEM image showing the SFs shearing γ″ phase; and (**h**,**i**) the corresponding HRTEM images of γ matrix and γ″ phase, respectively.

To provide a clearer distinction between slip bands and deformation twins, a more detailed microstructural characterization was performed on the creep-deformed and aged (3000 h) B2 alloy, as presented in Figure 13. HADDF and HRTEM images in Figure 13a–c indicate that the intragranular slip bands in the B2 alloy have a width of approximately 150 nm, while the deformation twins are relatively finer, with a thickness of about 30 nm. As illustrated in Figure 13d–f, the twins also distinctly shear the γ″ phase. Furthermore, dislocation tangles surrounding the γ″ phase are observed in regions near twin boundaries and slip bands, as indicated in Figure 13b,e. HRTEM observations of the intragranular γ″ phase showed that SFs primarily occur on the γ″ phase interface and propagate across it, with no continuous stacking faults detected in the matrix (Figure 13g–i). Observation of the creep mechanism in the B2 alloy reveals that, unlike the B0 alloy, the creep deformation mechanism of the B2 alloy involves the presence of slip bands in addition to twinning and shear faults. Some studies indicate that B segregation at grain boundaries promotes dislocation motion and slip, enhancing the plasticity of the alloy [17,30,33]. Therefore, B segregation at grain boundaries facilitates dislocation slip in adjacent grains, making the creep-deformed microstructure of the alloy more prone to the formation of dislocation slip bands.

In summary, under low-strain-rate creep tests, B significantly enhanced grain boundary strength, inhibiting grain boundary sliding and cavity nucleation, thereby extending the creep rupture life of the material. However, in rapid impact tests, due to the short loading time and high strain rate, the strengthening effect of B is not pronounced. These results indicate that the mechanism of B in superalloys is strongly dependent on diffusion processes, further confirming that the strengthening effect of B is closely related to temperature and time-dependent diffusion mechanisms.

## 4. Conclusions

This study examines the effect of B on the microstructural evolution of the IN718 superalloy during long-term aging. The distribution and underlying mechanisms of boron are elucidated by correlating room-temperature impact toughness and high-temperature creep properties across alloys with varying B contents. The main conclusions derived are as follows:

(1) B has little effect on the grain size of the alloy, but it suppresses the precipitation and coarsening of δ phase at grain boundaries during the aging process. In addition, B shows no significant influence on the coarsening of intragranular γ″ phase and the precipitation behavior of TCP phase during prolonged aging.

(2) B enhances the room-temperature impact toughness of alloys during the early stages of aging, and the improvement in impact toughness is primarily attributed to B segregation at grain boundaries, which strengthens intergranular cohesion. And the beneficial effect of B on mechanical properties stability was negligible during the later aging stages, as excessive grain boundary precipitation offset the enhancement in grain boundary cohesion provided by B segregation.

(3) An increase in B contents significantly enhances the creep stability of the alloy. This improvement is attributed to two primary mechanisms: first, B strengthens grain boundary cohesion, thereby suppressing the initiation and propagation of intergranular cracks; second, B restrains the coarsening of δ phase and suppresses the linkage and propagation of micro-cracks, substantially improving the stability of creep property.

(4) In the B-modified alloy, dislocation slip bands are more readily formed in the creep-deformed microstructure. As aging proceeds, the primary creep mechanism evolves from twinning and dislocation slip to a combination of twinning, stacking fault shearing, and dislocation slip.

## Data Availability

The original contributions presented in this study are included in the article. Further inquiries can be directed to the corresponding authors.
